# Regulators of Androgen Action Resource: a one-stop shop for the comprehensive study of androgen receptor action

**DOI:** 10.1093/database/bav125

**Published:** 2016-02-14

**Authors:** Adam D. DePriest, Michael V. Fiandalo, Simon Schlanger, Frederike Heemers, James L. Mohler, Song Liu, Hannelore V. Heemers

**Affiliations:** ^1^Department of Cancer Genetics; ^2^Department of Urology, Roswell Park Cancer Institute, Buffalo, NY, USA; ^3^Department of Cancer Biology, Cleveland Clinic, Cleveland, OH, USA; ^4^Department of Biostatistics and Bioinformatics, Roswell Park Cancer Institute, Buffalo, NY, USA; ^5^Department of Urology; ^6^Department of Hematology/Medical Oncology, Cleveland Clinic, Cleveland, OH, USA

## Abstract

Androgen receptor (AR) is a ligand-activated transcription factor that is the main target for treatment of non-organ-confined prostate cancer (CaP). Failure of life-prolonging AR-targeting androgen deprivation therapy is due to flexibility in steroidogenic pathways that control intracrine androgen levels and variability in the AR transcriptional output. Androgen biosynthesis enzymes, androgen transporters and AR-associated coregulators are attractive novel CaP treatment targets. These proteins, however, are characterized by multiple transcript variants and isoforms, are subject to genomic alterations, and are differentially expressed among CaPs. Determining their therapeutic potential requires evaluation of extensive, diverse datasets that are dispersed over multiple databases, websites and literature reports. Mining and integrating these datasets are cumbersome, time-consuming tasks and provide only snapshots of relevant information. To overcome this impediment to effective, efficient study of AR and potential drug targets, we developed the Regulators of Androgen Action Resource (RAAR), a non-redundant, curated and user-friendly searchable web interface. RAAR centralizes information on gene function, clinical relevance, and resources for 55 genes that encode proteins involved in biosynthesis, metabolism and transport of androgens and for 274 AR-associated coregulator genes. Data in RAAR are organized in two levels: (i) Information pertaining to production of androgens is contained in a ‘pre-receptor level’ database, and coregulator gene information is provided in a ‘post-receptor level’ database, and (ii) an ‘other resources’ database contains links to additional databases that are complementary to and useful to pursue further the information provided in RAAR. For each of its 329 entries, RAAR provides access to more than 20 well-curated publicly available databases, and thus, access to thousands of data points. Hyperlinks provide direct access to gene-specific entries in the respective database(s). RAAR is a novel, freely available resource that provides fast, reliable and easy access to integrated information that is needed to develop alternative CaP therapies.

**Database URL**: http://www.lerner.ccf.org/cancerbio/heemers/RAAR/search/

## Introduction

The androgen receptor (AR) is a member of the ligand-activated family of nuclear receptors. Upon activation by androgens, AR binds to androgen response elements (AREs) where it assembles the transcriptional complexes to control the expression of target genes (1, 2). The action of ligand-activated AR is critical for the progression of prostate cancer (CaP) to the lethal stage. Preventing AR activation and transcriptional activity through androgen deprivation therapy (ADT) remains the default treatment for locally advanced or metastatic CaP that cannot be cured by surgery or radiation (3, 4). CaP response to ADT is, with few exceptions, predictable: initial remission is followed by recurrence of CaP that remains dependent on AR signaling. Failure of ADT contributes directly to the ∼30000 CaP deaths in the US annually (5). Recurrence during ADT is associated with adaptive responses at different levels in the AR signaling axis (6–10). Some alterations occur at the level of AR itself and include AR overexpression, gain-of-function AR mutations or emergence of ligand-independent AR variants. Other adaptations affect the pre-receptor level of AR signal transduction, which entails the production of the bioactive ligand that activates AR, dihydrotestosterone (DHT), while still others affect the post-receptor level, which consists of a variety of molecular mechanisms by which activated AR to induce changes in target gene expression (9, 11). The latter two levels of the AR signaling axis may provide novel, indirect opportunities to target AR action and bypass the adaptive responses associated with conventional ADT.

**Figure 1.**
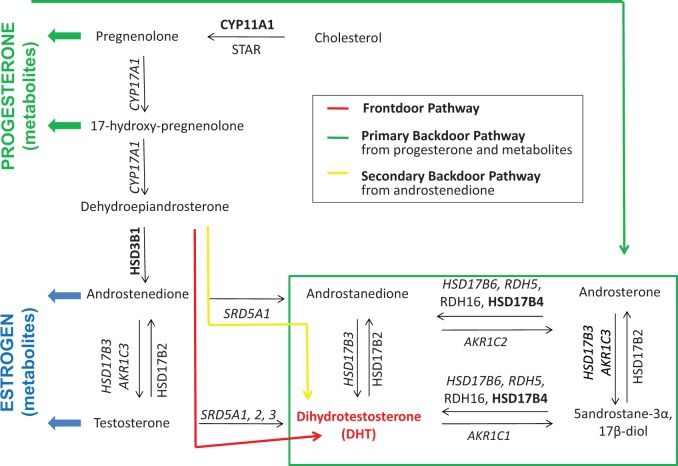
Flexibility in intraCaP DHT biosynthesis pathways. Schematic of principal androgen biosynthesis pathways and core enzymes that lead to DHT production in CaP cells. *Italicized text*, deregulated gene expression under ADT; boldface text, genomic alterations under ADT. Note that proteins involved in androgen transport and androgen degradation are not considered.

At the prereceptor level, efforts focus on targeting the key cellular events that control androgen biosynthesis in CaP cells (6, 12, 13). It has recently been discovered that, under selective pressure of combined testicular and adrenal ADT, CaP cells can control their DHT content due to flexibility in androgen biosynthesis pathways. Genomic and biochemical analyses of clinical CaP samples obtained before and after ADT have identified ‘primary backdoor’ pathways and ‘alternative backdoor’ pathways of androgen biosynthesis, in addition to the traditional ‘frontdoor’ pathway of androgen synthesis (Figure 1) (6, 12, 13). These pathways either convert alternative substrates into DHT, or skip steps that are essential for flux through the frontdoor pathway of DHT synthesis (Figure 1), and harbor steroidogenic enzymes that are gaining momentum as potential novel therapeutic targets (14, 15). In response to specific forms of ADT, adaptation occurs in the flux of androgen precursors and metabolites through these pathways, which presents potential new therapeutic targets. Changes include compensation in the expression levels or genomic alterations of genes involved in the synthesis, degradation or transport of androgens and/or accumulation of unused substrate that can be shunted into alternative biosynthetic pathways, resulting in, e.g. activation of mutant AR (16–20). These adaptations add to the well-recognized conversion of androgens to estrogens and the estrogen receptor’s role in CaP (21). In another alternative mechanism, glucocorticoids that alleviate side effects of ADT may activate the glucocorticoid receptor, which can then hijack the transcriptional program that is otherwise under AR control (22–24).

At the post-receptor level, the characterization of the AR cistrome and AR-dependent transcriptome has facilitated recognition of gene-specific contribution of coregulators and secondary transcription factors to AR-dependent transcription (8, 25–27). Targeting select components of AR transcription complex(es) may thus allow selective interference with a segment of AR action (9, 28). Coregulators in particular have long been viewed as attractive therapeutic targets for CaP treatment (29, 30). Expression of 50 coregulators is deregulated in CaP. Such deregulation often correlates with aggressive disease and is one of the mechanisms by which AR bypasses ADT (30, 31). Crude inhibitors for some coregulators are available already (32, 33), while Chem-Seq assays have isolated novel coregulator inhibitors and peptidomimetics now allow disruption of select AR-coregulator interactions (34, 35). Under the selective pressure of ADT, changes occur in coregulator function that contribute to AR’s sustained activity in CaP. ADT directly contributes to overexpression of androgen-regulated coregulator genes (25, 36–38), cellular redistribution of other coregulators that affect their interaction with AR (39, 40) and emergence of post-translational modifications that alter how coregulators influence AR-dependent transcription of subsets of genes (41). Specificity in coregulator contribution to gene expression may underlie at least in part a shift in the transcriptional program under control of AR in recurrent CaP (42, 43). It is important to consider also that the AR-dependent transcriptome between model systems and clinical CaP do not overlap completely (43).

## Database generation and its content

Renewed insights into the mechanisms by which CaP recurs despite ADT are uncovering novel approaches for therapeutic intervention at the AR pre-receptor and post-receptor levels (6, 9). The steroidogenic enzymes that are pursued for therapy now, and the signaling executed by androgen-activated AR represent only a fraction of the therapeutic potential. Determining, prioritizing and developing the true potential of novel targets requires that multidisciplinary data be taken into account. A wealth of information to facilitate these studies is available already in the public domain. Unfortunately, these data are stored in individual and disparate websites, databases and literature reports, making them difficult to locate and use.

To overcome this barrier to the study of AR in CaP, we have developed a novel tool, the Regulators of Androgen Action Resource (RAAR) database. The goal of RAAR is to accrue and provide information in a user-friendly and freely available manner to facilitate the development of novel therapies to inhibit AR action in CaP.

## Database content

RAAR gives access to information at two levels. First, it provides an up-to-date database of genes involved in pre-receptor and post-receptor regulation of AR signaling axis activity. Second, RAAR integrates access to well-established and well-curated databases that provide user-friendly and ready-to-use comprehensive information on gene function, clinical relevance, and technical details for research resources for each RAAR entry. Currently, RAAR contains entries for 329 genes involved in AR action in CaP and links to > 30 databases.

### RAAR as a database

RAAR gene entries are classified according to the function for which the gene is known, either pre-receptor or post-receptor. To date, the scenario in which a gene can be entered under both the pre-receptor and post-receptor sections applies only to the AKR1C3 gene. A wealth of reports substantiate AKR1C3’s involvement in multiple intracrine androgen biosynthesis pathways (12), but only one study has described AKR1C3 as a coregulator (44). As a consequence, AKR1C3 is included in the pre-receptor part of RAAR.

#### Pre-receptor level

An extensive literature review was done in PubMed searching for papers that mention in the title or abstract ‘AR’, ‘CaP’ and ‘ADT’ in combination with one or more of the following search terms ‘androgen synthesis’, ‘androgen metabolism’ and ‘androgen transport’. After review of the corresponding full-length manuscripts for any abstracts returned using these search terms, an overview was compiled of genes that encode proteins with critical roles in the uptake, biosynthesis and degradation of androgens and their precursors in CaP cells. The majority of RAAR entries relate to genes involved in enzymatic conversion of androgen. This includes the steroidogenic enzymes that are involved in the frontdoor, primary backdoor and secondary backdoor pathways of androgen biosynthesis (Figure 1). Androgens are derived from cholesterol, and some evidence indicates that the cholesterol biosynthetic pathway, the activity of which is upregulated in CaP (45), may participate in intraprostatic *de novo* synthesis of DHT, which starts from acetate (46). Because cholesterol has diverse roles in (cancer) cell biology, and lacks specificity to androgen biosynthesis, enzymes involved in cholesterol synthesis have not been included in RAAR. The steroidogenic acute regulatory protein (STAR), which contributes to the conversion of cholesterol to pregnenolone, is the most upstream component of androgen biosynthesis pathway that is included in RAAR (Figure 1). In view of the intraprostatic conversion of androgen and its precursors into estrogens and progestins, pivotal enzymes that catalyze the flux of androgen metabolites to and through these alternate steroid metabolic pathways are listed also. In recognition that glucocorticoids frequently are coadministered with ADT, key enzymes involved in glucocorticoid metabolism are incorporated. Proteins that mediate cellular import and export of androgens may affect further intraprostatic DHT levels, so members of the SLCO and ABC families of transporters that have been implicated in shuttling androgens across the cell membrane are listed in the pre-receptor section (47). Finally, the gene encoding sex hormone-binding globulin (SHBG) is included as it plays important roles in controlling free circulating testosterone levels and has roles within CaP cells as well (48). Currently, the RAAR pre-receptor dataset contains 55 entries.

#### Post-receptor level

A PubMed search for papers that contain the terms ‘AR’ and ‘CaP’ in their title and/or abstract was performed. As before (2), abstracts fulfilling these criteria were screened for reference to coregulator function, and if so, full-length papers were reviewed individually to verify description of a *bona fide* AR-associated coregulator. Following this review, 274 entries for coregulators that have been reported to modulate AR’s transcriptional output were included in the post-receptor level side of RAAR. This figure represents a substantial increase in the number of AR-associated coregulators (*n* = 170) compared to our previously reported inventory (2). Although several transcription factors contribute to ARE-driven transcription through functional or physical interaction with DNA-bound AR, cis-acting transcription factors are not included in RAAR. The mechanisms by which an individual transcription factor, for instance HoxB13, affects AR-dependent transcription are diverse and can entail both cis-action through binding to consensus binding motifs in close proximity of ARE(s) or recruitment as a cofactor to AR that does not involve binding to specific DNA recognition sequences (49). Transcription factors with roles as pioneering factors (e.g. FoXA1), impact also the genomic positions at which AR binds and are involved in the distribution of the AR-dependent cistrome (22, 50). These characteristics render transcription factors less attractive and less convenient targets for therapeutic intervention than coregulators, as discussed above in the Introduction. Cis-acting AR-interacting transcription factors are, therefore, not included in RAAR. We have opted to forego the traditional classification of coregulators as either coactivators or corepressors. Such grouping is becoming outdated rapidly by a growing body of evidence that a given coregulator enhances or diminishes the transcription of target genes in a context-dependent manner (25, 27, 51), which is consistent with the modular and variable composition and assembly of the coregulator complexosome (52).

### RAAR links to gene-specific data and other resources

For each gene entry at the pre- and post-receptor levels, RAAR provides hyperlinks to external databases containing information on gene function, clinical relevance and research resources. In addition, searches can be conducted by database through the RAAR tab ‘other resources’, which provides access to additional complementary datasets (Table 1).

**Table 1.** Overview of RAAR content

RAAR section	Entries (number)	Hyperlinked databases (number)	# Data points
Pre-receptor level	55	24	>1000/entry
Post-receptor level	274	25	>1000/entry
Other resources	36	36	>1000/entry

#### Pre-receptor and post-receptor levels

RAAR provides, for each of its 329 entries, centralized access to entry-specific information on gene function, clinical relevance and availability of research resources. This entry-specific information is maintained in 25 functionally diverse and well-curated external research databases that are freely accessible but scattered throughout the public domain. Table 2 provides an overview of the databases that can be accessed through RAAR. Inclusion of the NURSA database is limited to the RAAR post-receptor level entries as NURSA does not contain information that is relevant to the study of genes that are involved in steroid synthesis and transport. Information pertaining to gene function, clinical relevance and available research resources is further subclassified. For instance, the overarching category of gene function includes, but is not limited to, databases that list basic gene information, as well as those that report on gene structure, gene function, regulation of gene expression and interactome (protein or otherwise). The ‘clinical relevance’ cluster of databases encompasses those that allow the user to evaluate correlation of any of the gene data, or data on gene expression, genomic alterations and availability of drugs, with clinical outcome or therapeutic actionability. The resources section of Table 2 refers to those databases that provide information on the availability of a diverse array of reagents, model systems and assays to perform wet lab studies or assess further the clinical relevance of a selected RAAR entry, including references for relevant literature reports.

**Table 2.** Overview of databases to which RAAR provides entry-specific access and the types of information they provide

Database	URL	Gene function					Clinical relevance		Resources	
		Basic gene info	Gene structure	Gene functionality	Regulation of GEX	Interactome	Disease	Therapeutics	Reagents	References
Ensembl	http://www.ensembl.org	x	x	x	x					**x**
HGNC	http://www.genenames.org	x								**x**
APPRIS	http://appris.bioinfo.cnio.es	x	x	x						
COSMIC	http://cancer.sanger.ac.uk/cancergenome/projects/cosmic	x	x							**x**
ENCODE	http://www.genome.ucsc.edu/ENCODE	x	x	x	x		x			**x**
NCBI Gene	http://www.ncbi.nlm.nih.gov/gene	x	x			x				**x**
NCBI Nucleotide	http://www.ncbi.nlm.nih.gov/nuccore	x	x	x						**x**
NCBI Protein	http://www.ncbi.nlm.nih.gov/protein	x	x	x						**x**
UniProt	http://www.uniprot.org	x		x		x	x			**x**
ProteinDataBank	http://www.rcsb.org	x	x	x		x				**x**
Reactome	http://www.reactome.org			x		x				
neXtprot	http://www.nextprot.org		x	x		x	x			**x**
Domain mapping mutations	http://bioinf.umbc.edu/dmdm			x			x			
Atlas (Cyto)genetics	http://atlasgeneticsoncology.org/index.html	x	x			x	x			**x**
GenAtlas	http://genatlas.medecine.univ-paris5.fr/google/index_alx.php	x	x	x	x		x			
Oncomine	https://www.oncomine.org				x		x			**x**
cBioPortal	http://www.cbioportal.org/public-portal		x		x	x	x			
Medicalgenomics	http://www.medicalgenomics.org	x			x	x				
NCBI dbSNP	http://www.ncbi.nlm.nih.gov/projects/SNP		x	x			x			**x**
SNPper	http://snpper.chip.org/		x	x						**x**
PharmGKB	http://pharmgkb.org	x					x	x		**x**
GeneProf	http://geneprof.org		x	x	x	x	x			
NURSA	http://www.nursa.org	x			x	x	x			**x**
GeneCards	http://www.genecards.org/	x	x	x	x	x	x	x	x	**x**
Labome	http://www.labome.com								x	

**Figure 2.**
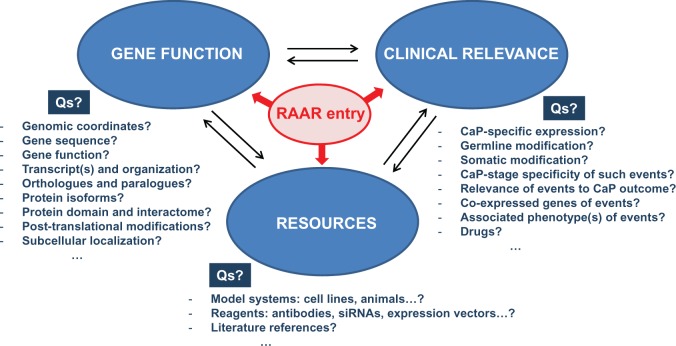
Sample of the information that RAAR provides for each of its entries. For each entry, RAAR allows cycling between information on different aspects of gene function, clinical relevance and availability of resources. Examples of representative questions (Qs) that RAAR answers are also shown.

Although individual databases provide specialized data with an emphasis on one or more aspects of the above described subclassifications, information is typically not restricted to the emphasized aspects. Consequently, data will overlap between databases for a given RAAR entry, but such overlap will enable the user to make a more informed selection of which RAAR entries to pursue to identify potential targets for therapeutic intervention. The RAAR set-up allows users to collect thousands of diverse pieces of data on each entry. By unifying and colocalizing such an unprecedented level of information, RAAR provides the opportunity for an investigator to integrate data on a broad spectrum of topics as diverse as the chromosomal location of the gene of interest, intron/exon gene organization, pseudogenes, genomic alterations and the functional consequences of such alterations, the protein interactome, clinical relevance of gene expression in CaP as well as technical details for antibodies, primers, activity assays, siRNAs, drugs and druggability, etc. … Figure 2 provides an overview of the type of questions that RAAR can answer in an integrated manner in the pre- and post-receptor level databases.

#### Additional resources level

In addition to databases included for the pre- and post-receptor data, RAAR provides access to other well-known and well-maintained databases. We decided to include these other resources for various reasons. For some of these databases (e.g. rSNPBase, canSAR) a gene-specific entry (see below) could not be established in the pre- or post-receptor sections, yet the resource itself is relevant for study of the gene of interest listed in RAAR or may give more detail on entry-specific insights derived from the pre-receptor or post-receptor section. Information maintained in other databases is complementary but not identical to what is provided already in RAAR (e.g. the AR mutational database). Links to databases providing specific tools that allow one to compare and analyze genes and gene signatures and regulatory DNA sequences in more detail (e.g. Galaxy, Cistrome, Blast) are included also. A link to Nucleic Acid Research’s Molecular Biology Databases, several of which may be helpful in enhancing the value of RAAR-derived insights, and links to homepages of the databases listed in Table 2 are included also.

## User interface

**Figure 3.**
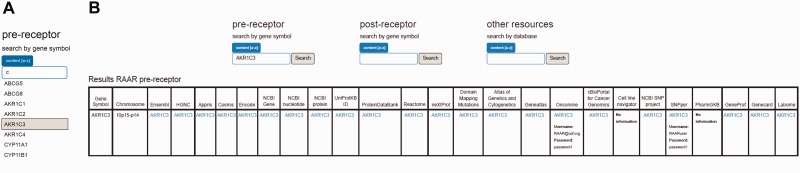
Screenshot of autocomplete function that lists eligible search terms (**A**) and basic RAAR search form and sample query result (**B**) for pre-receptor database.

**Figure 4.**
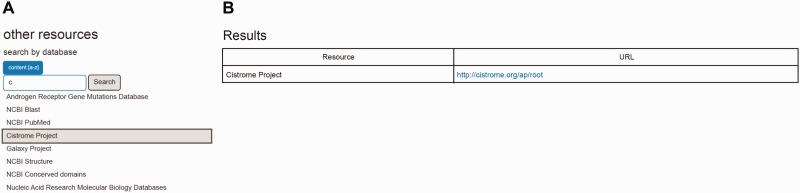
Screenshot of autocomplete function that lists eligible search terms (**A**) and basic RAAR search form and sample query result (**B**) for other resources database.

RAAR is set up as a searchable web interface (http://www.lerner.ccf.org/cancerbio/heemers/RAAR/search/) that is accessible also via a link on the senior author’s (H.V. Heemers) laboratory web site (http://www.lerner.ccf.org/cancerbio/heemers/). Gene lists of critical regulators of intra-CaP androgen biosynthesis and AR-associated coregulators were converted into CSV format files, which were used as input for a MySQL database. Database access is provided by a user interface in PHP and AJAX and a web interface that runs on a Linux (2.6.18)—Apache (2.0) MySQL (5.0.77)—PHP (5.3.6) platform. The MyISAM database storage engine manages non-transactional tables, provides high-speed storage and retrieval and supports full-text searching. The search interface allows searching the pre-receptor and the post-receptor databases by official gene symbol; the other resources database is searched by database name (Figure 3b). The search term can be entered directly into the RAAR search fields or can be selected from the dropdown list when one clicks the ‘Content [a-z]’ tab. An auto-complete function (Figures 3a and 4a) that lists eligible search terms containing the entered string at any position is available also. The ‘Search’ button launches the query and returns the search results. Figures 3b and 4b show the basic search form and sample query results.

An introduction to RAAR that familiarizes the user with the organization of the information and a user manual are included on the webpage. None of the information to which RAAR provides access is proprietary; all of it can be consulted by users searching the public domain. The RAAR content is made freely accessible to the research community and is not password-protected. The Oncomine and SNPper databases are included but, per their requirements, require the user to log in. Any academic or non-profit user can establish a free log-in account by completing a simple registration process. For RAAR users who are not yet registered, RAAR has included a log-in and password in the RAAR user manual. This log-in information is included in RAAR query results also (Figure 3b).

## Database implementation

All RAAR entries are entered using their official gene symbol as designated by the HUGO gene nomenclature committee. Access to aliases for RAAR entries can be obtained through the Gene Entry database of the resource. RAAR entries are listed in alphabetical order in the pre-receptor and post-receptor tabs, and the order in which the individual databases are listed is shown in Table 2. This database order reflects grouping based on database content: gene function, clinical relevance and research resources. As mentioned above, access to NURSA is provided in the post-receptor level RAAR tab only. For each RAAR entry, access to the >20 databases is given through hyperlinks that take the user to entry-specific information. The use of hyperlinks required selecting specific aspects of the database pages to which the RAAR entries lead. This choice was governed by the organization and level of information provided in the respective databases. In some cases (e.g. GeneCard), the user is taken to the page that provides an overview of all the information the database contains for that RAAR entry. On other occasions, for instance cBioPortal, the user is taken to CaP-specific information for RAAR entries. How a specific database was designed also had to be considered in selecting the specific site to which RAAR hyperlink leads. With cBioPortal, we opted to lead the user to a view of entry-specific copy number variation and somatic mutation data from all available CaP studies (which include all available TCGA CaP data). We chose this option because the user can see at a glance that the particular information presented on this page complements the views of CaP-specific gene expression and outlier data in Oncomine and germline mutation data in SNPper. This approach also allows the user, with minimal effort, to scroll back in cBioPortal to access gene co-expression patterns from same patient cohort.

Some databases return multiple hits when searched for RAAR entries using their official HUGO gene symbol. This applies for instance to the NCBI Nucleotide and NCBI Protein database, which contain sequences and other information on multiple gene transcripts and isoforms. In those cases, RAAR is set up so the hyperlink takes the user to the ‘standard reference’, which is usually the transcript or isoform version that corresponds to the longest coding sequence. The organization of RAAR gives the user the opportunity to perform further searches within these databases and/or compare these results with others within relevant RAAR resources (e.g. ENCODE).

For some RAAR entries, no content is available in the selected external databases. In those cases, RAAR includes no hyperlink for that entry, which is reflected in a ‘no information’ statement on the results page. Hyperlinks for these RAAR entries will be added as the corresponding database information becomes available.

RAAR content is curated manually by Heemers lab members every 3 months. The content of the external databases to which hyperlinked access is provided is monitored frequently. In the event that major updates or additions to external databases occur prior to scheduled RAAR maintenance, RAAR will be updated at an accelerated schedule.

RAAR strives to be a resource that provides fast, reliable and easy access to integrated information on key regulators of AR action. Recommendations for updates, improvements and additions are welcome. Readers and users are encouraged to forward their suggestions to RAAR@ccf.org.

## Discussion

Improving ADT so that treatment is biomarker-driven and individualized requires complementary expertise in molecular biology, genomics, clinical urology and bioinformatics. RAAR provides not only an up-to-date overview of key regulators of AR activation and transcriptional activity, but brings together in one place an array of diverse resources to facilitate access to multidisciplinary information via simple, user-friendly searches. An advantage of the compendium side of RAAR is it the ease with which the user can study simultaneously the co-operativity, or redundancy, between genes in RAAR. This is an important consideration in view of compensatory mechanisms that govern the activity of both steroidogenic enzymes and coregulators. At the molecular level, RAAR allows the user to, for instance, identify and compare easily the catalytic sites that are present in different steroidogenic enzymes. From a translational perspective, the RAAR user can determine co-expression patterns of regulators and downstream effectors of AR action, correlate differential expression with CaP outcome and survival, and verify the CaP-specificity of these events. For example, merging molecular and clinical aspects, RAAR allows one to evaluate the impact of a genomic alteration that is associated with clinical CaP progression on its protein function, protein interactome and technical resources that are available to pursue the observation further at the bench.

The topic of RAAR, namely the genomic action of a nuclear receptor, is similar to, but does not overlap with, other publicly available resources, such as those included in Nucleic Acids Research’s Molecular Biology Database Collection. The AR mutation database (53), which provides a comprehensive overview of mutations within the AR gene, is closest in nature and scope to RAAR. The AR mutation database gives a succinct overview of an incomplete subset of AR-interacting transcription factors and coregulators, such that it is limited to a list of these proteins and their AR interaction site. To avoid overlap with the AR mutation database, we did not include AR as an entry in RAAR, and thus, the two databases complete and complement each other, rather than compete with one another. NURSA (54) provides an array of data on different aspects of general coregulator function, but as reflected in RAAR’s post-receptor level section, the overlap between NURSA’s content and that of RAAR is surprisingly limited. As a consequence, the information provided by RAAR is relevant also to those investigators who study the molecular contribution of coregulators to transcription factor action in general. RAAR’s focus on CaP and AR means that the vast majority of information is RAAR-specific for those coregulators that are present in both RAAR and NURSA.

RAAR was established by CaP researchers seeking to understand better and target more efficiently AR action in this endocrine cancer. The wealth of diverse data on AR action that is included also makes RAAR a valuable resource for investigators who study other human conditions in which AR is a viable but poorly understood target for therapy. For instance, AR is starting to be recognized as attractive target in triple-negative (ER-/PR-/Her2-) breast cancer (55) and may be a critical target in other human malignancies in which sex differences exist in incidence and mortality, such as bladder cancer and hepatic cell carcinoma (56, 57). At the other end of the spectrum, defects at both pre- and post-receptor levels of the AR signaling axis are involved in partial or complete androgen insensitivity syndromes (58, 59). RAAR may also be useful for researchers who seek to develop treatments to reactivate AR signaling, which is the goal for therapy in individuals affected by these syndromes. In a broader perspective, because of the nature and scope of information it provides, RAAR facilitates in an unprecedented and comprehensive manner the study of steroid-activated nuclear receptors in general.
